# 

**Published:** 1927

**Authors:** 


### The Bristol Medico-Chirurgical Journal

A JOURNAL OF THE MEDICAL SCIENCES FOR THE WEST OF ENGLAND AND SOUTH WALES.

PUBLISHED UNDER THE AUSPICES OF

*THE BRISTOL MEDICO-CHIRURGICAL SOCIETY.*

EDITOR:

J. A. NIXON, C.M.G., M.D., F.R.C.P.

WITH WHOM ARE ASSOCIATED

E. W. HEY GROVES, M.S., F.R.C.S., B.Sc.

E. WATSON-WILLIAMS, M.C., Ch.M., F.R.C.S.E.

A. E. ILES, O.B.E., M.B., B.S. Lond., F.R.C.S.

CAREY F. COOMBS, M.D., F.R.C.P., Assistant Editor.

EDITORIAL SECRETARY:

A. L. FLEMMING, M.B., B.Ch.

---

"Scire est nescire, nisi id me

Scire alius scierit."

---

*VOL. XLIV.*

BRISTOL · J. W. ARROWSMITH LTD.
London: J. W. Arrowsmith (London) Ltd.
1927.

ARMY MEDICAL LIBRARY

296936

WASHINGTON, D.C.

WI
BR323

### Contents of Vol. XLIV.

*Page*

Anæsthesia. By A. L. Flemming, M.B., B.Ch. .. .. .. .. 1

The Recommendations of the Royal Commission on Lunacy and Mental Disorder. By J. V. Blachford, C.B.E., M.D. .. .. .. 15

The Central Nervous System in Addisonian Anæmia. By Geoffrey Hadfield, M.D., M.R.C.P. Lond... .. .. .. .. .. 21

Observations on Pellagra. By E. Barton White, M.R.C.S. Eng., L.R.C.P. Lond., and Geoffrey Hadfield, M.D., M.R.C.P. Lond. .. .. 31

Some Clinical Aspects of Sterility. By R. S. S. Statham, O.B.E., M.D., Ch.M. .. .. .. .. .. .. .. .. .. .. 41

Early Medical Teaching in Bristol. By F. Richardson Cross, M.B. Lond., LL.D. Bristol, F.R.C.S. .. .. .. .. .. .. .. 73

The Endometriomata. By A. D. Fraser, M.A., B.Sc., M.B. .. .. 113

The Significance of Vertigo. By E. Watson-Williams, M.C., Ch.M., F.R.C.S. Ed. .. .. .. .. .. .. .. .. .. 119

The Prophylaxis of Ophthalmia Neonatorum. By A. E. Iles, O.B.E., M.B., F.R.C.S. Eng. .. .. .. .. .. .. .. .. 125

The Long Fox Memorial Lecture: Some Considerations on the Cells and Liquids of the Body and the Oxygen-content of these. By George A. Buckmaster, M.A., M.D. Oxon. .. .. .. .. .. .. 153

A Visit to the Near East. By George Parker, M.D. Cantab. .. .. 173

Some Clinical Aspects of Dysmenorrhœa. By R. S. S. Statham, O.B.E., M.D., Ch.M. .. .. .. .. .. .. .. .. .. 187

Six Cases of Cancer arising in Tissues within the Duodenal Loop. By Helen B. Murgoci .. .. .. .. .. .. .. .. 195

The Hospitals of Madrid: A "Busman's Holiday." By E. W. Hey Groves, M.S., F.R.C.S. .. .. .. .. .. .. .. .. 229

Acute Suffocative Œdema of the Lungs. By J. Anthony Birrell, M.D., M.R.C.P. .. .. .. .. .. .. .. .. .. 239

Coronary Obstruction. By Carey F. Coombs, M.D., F.R.C.P. .. .. 249

The Pathology of Coronary Occlusion. By Geoffrey Hadfield, M.D., M.R.C.P. .. .. .. .. .. .. .. .. .. 257

Two Cases of Angina Pectoris following Sepsis. By Newman Neild, M.B. Vict., M.R.C.P. .. .. .. .. .. .. .. 263

The Health of the Nation—A Review .. .. .. .. .. 267

Reviews of Books .. .. .. .. .. .. 49, 129, 209, 269

Editorial Notes .. .. .. .. .. .. .. 57, 138, 221, 274

Meetings of Societies .. .. .. .. .. .. 59, 141, 223, 277

Obituary .. .. .. .. .. .. .. .. .. .. .. 61

The Medical Library of the University of Bristol .. 62, 148, 225, 281

Local Medical Notes .. .. .. .. .. .. 64, 151, 228, 283

### The Medical Library of the University of Bristol,

WITH WHICH IS INCORPORATED

**The Library of the Bristol Medico-Chirurgical Society.**

*The following donations have been received since the publication of the List in December,* 1926.

January, 1927.

| | | |
|---|---|---|
| Dr. W. S. Bainbridge (1) .. .. .. | 1 | volume. |
| Professor Edward Fawcett (2) .. .. | 1 | ,, |
| Professor E. W. Hey Groves (3) .. .. | 5 | volumes. |
| Michell Clarke Memorial Fund (4) .. .. | 13 | ,, |
| The Rockefeller Foundation (5) .. .. | 1 | volume. |
| The Royal College of Physicians of Edinburgh (6) | 1 | ,, |

Unbound periodicals have also been received from Professor E. W. Hey Groves.

### THE ONE HUNDRED AND NINETEENTH LIST OF BOOKS.

The figures in round brackets refer to the figures after the names of the donors. The books to which no such figures are attached have either been bought from the Library Fund or received through the *Journal.*

**Blumberg, J.** .. .. *Lehrbuch der Topographischen Anatomie* .. (3) 1926
**Bruce, J. M. and Dilling, W. J.** *Materia Medica and Therapeutics* 13th Ed. 1926
**Cameron, S. J. and Hewitt, J.** *Difficult Labour* .. .. .. .. .. .. .. 1926
**Cumston, C. G.** .. .. *History of Medicine* .. .. .. .. .. .. .. 1926
**Dupont, R., Leroux, R., and Dalsace, J.** *Prélèvements et des Biopsies* (3) 1926
**Fifield, L. R.** .. .. *Infections of the Hand* .. .. .. .. .. .. 1926
*Historical Sketch and Laws of the Royal College of Physicians, Edinburgh* (6) 1925
**Hutchison, R.** .. .. *Elements of Medical Treatment* .. .. .. .. 1926
**Jack, W. R.** .. .. *Wheeler's Handbook of Medicine* .. .. 8th Ed. 1927
**Kirchberg, Fr.** .. .. *Massage und Gymnastik* .. .. .. .. .. .. (3) 1926
**Lenk, R.** .. .. .. *Index and Handbook of X-ray Therapy* .. .. 1926
**Love, R. J. M.** .. .. *A Shorter Surgery* .. .. .. .. .. .. .. .. 1926
**McLaughlin, A. I. G.** *Diseases of the Heart and Lungs* .. .. .. .. 1926
**Price, F. W.** .. .. *Diseases of the Heart* .. .. .. .. .. 2nd Ed. 1927
**Rollier, R.** .. .. .. *Heliotherapy* .. .. .. .. .. .. .. 2nd Ed. 1927
**Sanderson, W. and Rayner, E. B. A.** *Introduction to Law and Tradition of Medical Practice* .. .. .. .. .. .. .. .. 1926
**Scudder, C. L.** .. .. *Treatment of Fractures* .. .. .. 10th Ed. (3) 1926
**Treves, F.** .. .. .. *Surgical Applied Anatomy* .. .. .. 8th Ed. 1926
**Troup, W. A.** .. .. *Ultra-violet Rays in General Practice* .. .. .. 1926
**Wenyon, C. M.** .. .. *Protozoology.* 2 Vols. .. .. .. .. .. .. .. 1926
**Woodwark, A. S.** .. *Manual of Medicine* .. .. .. .. 3rd Ed. 1927
**Woolf, M. S.** .. .. *Principles of Surgery for Nurses* .. .. .. (3) 1925

### TRANSACTIONS, REPORTS, JOURNALS, ETC.

American Journal of Hygiene (Monographic Series), No. 7, October (2) 1926
Annual Report. Rockefeller Foundation .. .. .. .. .. .. .. (5) 1925
Journal of Obstetrics and Gynæcology of the British Empire. Index XI-XXX .. .. .. .. .. .. .. .. .. .. .. .. .. .. .. 1926
Nouveau traité de mèdecine. Vols. I, IV, V, VI, VII, VIII, XI, XII, XIII, XIV, XV, XIX, XXII .. .. .. .. .. .. .. .. .. .. (4)
Progressive Medicine. Vol. IV.. .. .. .. .. .. .. .. .. .. .. .. 1926
Progressive Medicine. Vol. IV.. .. .. .. .. .. .. .. .. .. .. .. 1926
Report on 3rd Internat. Congress of Military Medicine and Pharmacy (1) 1926
St. Thomas's Hospital Reports. Vol. 48 .. .. .. .. .. .. .. .. 1926

### Local Medical Notes.

Dr. J. A. Nixon has been elected to the Council of the Royal College of Physicians, London.

Dr. Macdonald Critchley has been appointed Registrar at the National Hospital, Queen Square, London.

EXAMINATION RESULTS.

**University of Bristol.**—Students of the University have recently passed the following examinations :—

M.B., Ch.B. (*Part I.—Including Forensic Medicine and Toxicology. Pass :* T. H. Berrill (Distinction in Materia Medica, Pharmacy, Pharmacology and Therapeutics), B. J. Boulton, T. A. Brand, R. M. Norman (Distinction in Materia Medica, Pharmacy, Pharmacology and Therapeutics), E. Southam, T. B. Wansbrough (Distinction in Materia Medica, Pharmacy, Pharmacology and Therapeutics), A. R. Williams. (*Part I. only*). *Pass :* G. D. J. Ball, O. J. P. Bollon, T. L. Cleave, R. D. Jenkins, (Miss) H. B. Murgoci, (Miss) E. M. Redman.

M.B., Ch.B. (*Part II.*)—*Completing Examination. Pass :* (Miss) W. P. Brinckman, J. M. Camps-Campin, A. M. Critchley, T. F. Hewer (with Distinction in Pathology), F. W. T. Hughes, R. Jackson (with Distinction in Forensic Medicine and Toxicology), E. R. Wide. *Group I.* (*only*). (Miss) H. M. Aldwinckle.

B.D.S.—*Final Examination. Pass :* E. C. Browne. *Third Examination in Materia Medica* (*Completing Examination*). *Pass :* W. M. Evans.

Diploma in Dental Surgery.—*Final Professional Examination. Pass :* J. J. G. Bishop, P. H. Slater, P. J. Whiteman, A. E. Wilkins, C. S. Wilde.

Diploma in Dental Surgery.—*Second Professional Examination. Pass :* H. G. Clapp. *In Dental Mechanics* (*Completing Examination*) *:* A. L. Gilchrist. *In Dental Mechanics and Dental Metallurgy only :* C. R. H. Edwards, L. W. G. Jones, H. J. Phillips, (Miss) S. M. Weir.

M.R.C.S., L.R.C.P.—(Miss) W. P. Brinckman, F. H. Woolley.

L.S.A.—*Surgery. Section II. :* N. S. J. Roberts. *Midwifery :* S. K. Rigg.

### Bristol Medico-Chirurgical Society.

### President.

A. L. FLEMMING, M.B., B.Ch. Brist.

### President-Elect.

W. K. WILLS, O.B.E., M.A., M.B., B.Ch. Cantab.

### Committee.

*Ex-Officio*
- T. CARWARDINE, M.S. Lond. (Retiring President).
- J. A. NIXON, C.M.G., B.A., M.D.Cantab. (Editor of *Journal*).
- A. L. FLEMMING, M.B., Ch.B. Brist. (Editorial Secretary).
- W. A. SMITH, M.A., M.B. Oxon. (Hon. Librarian).

*Elected.*

F. LACE, F.R.C.S. } Ex-Presidents.
J. O. SYMES, M.D. Lond. } Ex-Presidents.
D. C. RAYNER, F.R.C.S.
C. FERRIER WALTERS, F.R.C.S.
E. W. HEY GROVES, M.D., M.S. Lond.
W. VINCENT WOOD, *M.C.*, B.A. Cantab.
H. L. ORMEROD, M.D. (R.U.I.).

### Honorary Secretary.

W. A. JACKMAN, M.B., F.R.C.S. Ed.

### Honorary Treasurer.

A. E. ILES, O.B.E., M.B., B.S., F.R.C.S.

Medical Practitioners wishing to join the Society are requested to communicate with the Hon. Secretary, Mr. W. A. JACKMAN, 15 Mortimer Road, Clifton. The Annual Subscription is £1 11s. 6d.. payable to Hon. Treasurer.

---

*Extracts from Journal By-laws.*

4.—The *Journal* shall be delivered free to Subscribers and also to Members of the Society whose subscriptions are not in arrear.

6.—The Annual Subscription to the *Journa* is 10/6, payable to Hon. Treasurer.

---

Communications intended for insertion in the *Journal* should be sent to the Editor, Dr. J. A. NIXON, 7 Lansdown Place, Clifton, Bristol.

Books for Review and Exchange Journals should be sent to the Assistant-Editor, *Bristol Medico-Chirurgical Journal*, Medical Library, The University, Bristol.

Communications referring to the delivery of the *Journal* should be sent to the Editorial Secretary, A. L. Flemming, 48 Pembroke Road, Clifton, Bristol.

Cases for Binding Vols. I—XLIII are now ready, and may be obtained, price 1/9 each (postage extra), upon application to J. W. ARROWSMITH LTD., Quay Street, Bristol; or the *Journal* will be bound. including Case, for 7/- per volume.

### Bristol Medico-Chirurgical Society.

### PAST PRESIDENTS.

1874—75. FREDERICK BRITTAN, M.D., B.A. Dub.
1875—76. ROBERT WILLIAM COE.
1876—77. SAMUEL MARTYN, M.D. Ed.
1877—78. AUGUSTIN PRICHARD, M.D. Berlin.
1878—79. HENRY EDWARD FRIPP, M.D. Würzburg.
1879—80. WILLIAM MICHELL CLARKE.
1880—81. JOSEPH GRIFFITHS SWAYNE, M.D. Lond.
1881—82. EDWARD LONG FOX, M.D. Oxon.
1882—83. JAMES GEORGE DAVEY, M.D. St. And.
1883—84. WILLIAM JOHNSTONE FYFFE, M.D., B.A. Dub.
1884—85. GEORGE FORSTER BURDER, M.D. Aberd.
1885—86. WILLIAM HENRY SPENCER, M.D., M.A. Cantab.
1886—87. FRANCIS POOLE LANSDOWN.
1887—88. LEMUEL MATTHEWS GRIFFITHS.
1888—89. ROBERT SHINGLETON SMITH, M.D., B.Sc. Lond.
1889—90. NELSON CONGREVE DOBSON, Ch.M. Brist.
1890—91. SAMUEL HENRY SWAYNE.
1891—92. FRANCIS RICHARDSON CROSS, M.B. Lond., LL.D. Brist.
1892—93. EDWARD MARKHAM SKERRITT, M.D., B.S., B.A. Lond.
1893—94. JAMES GREIG SMITH, M.B., C.M., M.A. Aberd., F.R.S.E.
1894—95. ALFRED JAMES HARRISON, M.B. Lond.
1895—96. ARTHUR WILLIAM PRICHARD.
1896—97. ALFRED EDWARD AUST LAWRENCE, M.D., C.M. Aberd.
1897—98. JOHN EDWARD SHAW, M.B., C.M. Ed.
1898—99. ROBERT ROXBURGH, M.B. Ed.
1899—00. WILLIAM HENRY HARSANT.
1900—01. DAVID SAMUEL DAVIES, M.D. Lond.
1901—02. BARCLAY JOSIAH BARON, M.B., C.M. Ed.
1902—03. GEORGE MUNRO SMITH, M.D. Brist.
1903—04. JAMES PAUL BUSH, C.M.G., C.B.E., Ch.M. Brist.
1904—05. REGINALD EAGER, M.D. Lond.
1905—06. JOHN DACRE.
1906—07. JAMES TAYLOR.
1907—08. HENRY WALDO, M.D., C.M. Aberd.
1908—09. JOHN MICHELL CLARKE, M.D., M.A. Cantab.
1909—10. JAMES SWAIN, C.B., C.B.E., M.D., M.S. Lond.
1910—11. BERTRAM MITFORD HERON ROGERS, M.D., B.Ch., B.A. Oxon.
1911—12. CHARLES ALEXANDER MORTON.
1912—13. WALTER CARLESS SWAYNE, M.D., B.S. Lond. and Brist.
1913—14. PATRICK WATSON-WILLIAMS, M.D. Lond.
1914—15. WILLIAM HARRY CHRISTOPHER NEWNHAM, M.A., M.B. Cantab.
1915—16 GEORGE PARKER, M.A., M.D. Cantab.
1916—17 FRANCIS HENRY EDGEWORTH, M.A., M.D. Cantab., D.Sc. Lond.
1917—18 FREDERICK LACE.
1918—19 ROBERT GUTHRIE POOLE LANSDOWN, M.D., B.S. Durh.
1919—20 I. WALKER HALL, M.D., Ch.B. Vict.
1920—21 LEOPOLD ERNEST VALENTINE EVERY-CLAYTON, M.D. Lond.
1921—22 CYRIL HUTCHINSON WALKER, M.B., B.A. Cantab.
1922—23 JOHN ALEXANDER NIXON, C.M.G., B.A., M.D. Cantab.
1923—24 JOHN LACY FIRTH, M.D., M.S. Lond.
1924—25 JOHN ODERY SYMES, M.D. Lond.
1925—26 THOMAS CARWARDINE, M.S. Lond.

1927.

### LIST OF SUBSCRIBERS

TO THE

### Bristol Medico-Chirurgical Journal.

### HONORARY MEMBERS OF THE SOCIETY.

| | |
|---|---|
| Swain, James, *C.B.*, *C.B.E.*, M.D., M.S. ... ... ... ... | Wyndham House, Easton-in-Gordano. |
| Watson-Williams, P., M.D.... | 2 Rodney Place, Clifton. |

### SUBSCRIBERS.

*Those marked * are Members of the Society.*

| | |
|---|---|
| *Adams, A. W., M.S.. Lond. ... ... ... ... | 9 Mortimer Road, Clifton. |
| Adye, W. J. H. ... ... ... ... ... ... ... ... | St. Margaret's, Bradford-on-Avon. |
| *Alexander, A., M.A. ... ... ... ... ... ... | 23 Manilla Road, Clifton. |
| *Alexander, D. A., M.B., Ch.B. Vict. ... ... | 30 Berkeley Square, Clifton. |
| Alexander, G. L., B.A. Cantab. ... ... ... | 30 Berkeley Square, Clifton. |
| Alford, H. J., M.D. ... ... ... ... ... ... ... | 4 Park Terrace, Taunton. |
| *Askins, R. A., M.D., B.Ch., B.A.O. Dub. ... | Guildhall, Bristol. |
| *Aubrey, T., M.B. Lond. ... ... ... ... ... | Bitton, near Bristol. |
| *Baker, Lily A., M.D., B.Ch., B.A.O., R.U.I.... | 21 Victoria Square, Clifton. |
| *Barber, M. C., M.B., Ch.B. Brist. ... ... ... | Ingledene, High Street, Staple Hill, Bristol. |
| *Barker, G. H., M.D., B.Sc. Lond. ... ... ... | 124 Redland Road, Bristol. |
| *Bartholomew, E. U. ... ... ... ... ... ... | 12 Lansdown Place, Clifton. |
| Beatson, D. H., M.B., Ch.B. ... ... ... ... | 8 Richmond Park Road, Clifton. |
| *Benson, Elizabeth, M.B., Ch.B. Brist. ... | 8 Theresa Place, Bristol Road, Gloucester. |
| *Bergin, F. G. ... ... ... ... ... ... ... ... | 31 Oakfield Road, Clifton. |
| Bernard, C. ... ... ... ... ... ... ... ... | 564 Fishponds Road, Fishponds, Bristol |
| Berry, F. C., M.D., B.Ch., B.A. Dub. ... ... | Locarno, Burnham, Somerset. |
| *Birrell, J. A., M.D., M.S. Lond. ... ... | 51 Sussex Place, Ashley Road, Bristol. |
| *Blachford, J. V., *C.B.E.*, M.D. Durh. ... ... | 1 Victoria Square, Clifton. |
| Blackett, J. F., M.D., B.S. Lond. ... ... ... | Hamsterley, Bloomfield Park, Bath. |
| *Blagg, Arthur F., M.D. Durh.... ... ... ... | 6 Upper Belgrave Road, Clifton. |
| *Blathwayt, A. de V.... ... ... ... ... ... | 6 Brock Street, Bath. |
| Bletchly, G. Playne, M.B. Lond. ... ... ... | Hazelwood, Nailsworth, Glos. |
| *Bodman, C. O., M.D. Durh. ... .. ... ... | 17 Upper Belgrave Road, Clifton. |
| *Bodman, F. H., M.B., Ch.B. Brist. ... ... ... | 170 Cheltenham Road, Bristol. |
| *Bodman, J. Hervey, M.D. Lond. ... ... ... | 9 Whiteladies Road, Clifton. |

| | |
|---|---|
| *Bowker, G. E., M.D. Ed. ... ... ... ... ... ... | 11 North Parade, Bath. |
| *Bradbeer, E. G., M.B., Ch.B. Brist. ... ... | 32 Linden Road, Westbury Park, Bristol. |
| *Bradley, W. H., B.M., B.Ch. Oxon. ... ... | St. Wulstan's, Stratton-on-Fosse, Bath. |
| *Bray, G., Lt.-Col., *D.S.O.* ... ... ... ... | 26 Beaconsfield Road, Clifton. |
| *Brentnall, T. C. ... ... ... ... ... ... ... ... | Bellfield, Brightlingsea, Essex. |
| *Britton, R. B., M.B., B.S. Lond. ... ... ... | Troy House, Kingswood, Bristol. |
| *Buckmaster, G. A., M.A., M.D. Oxon. ... ... | University of Bristol. |
| *Burdon-Cooper, J., M.D., B.Sc. Durh. ... | 12 The Circus, Bath. |
| *Burges, R. ... ... ... ... ... ... ... ... ... | Somerset House, Canynge Road, Clifton. |
| *Burnett, R. H., M.B., B.S. Durh. ... ... ... | 479 Bath Road, Brislington, Bristol. |
| *Bush, G. B., M.B., Ch.B. Brist. ... ... ... | 5 Lansdown Place, Clifton. |
| *Bush, J. Paul, *C.M.G.*, *C.B.E.*, Ch.M. Brist. | 29 Caledonia Place, Clifton. |

| | |
|---|---|
| *Cairns, F. J. ... ... ... ... ... ... ... ... | Devon House, Whitehall Road, Bristol. |
| *Carey, R. S., *O.B.E.*, B.A. Cantab. ... ... | 502 Bath Road, Brislington, Bristol. |
| *Carleton, H. H., M.A., M.D. B.Ch. Oxon. | 1 Lansdown Road, Clifton. |
| *Carling, A., M.A., M.B., B.C. Cantab. ... ... | 12 Great George Street, Bristol. |
| *Carter, T. M. *O.B.E.*, M.D. ... ... ... | 19 Westfield Park, Redland, Bristol. |
| *Carwardine, Thomas, M.B., M.S. Lond. ... | 16 Victoria Square, Clifton. |
| *Cave, Edward J., M.D. Lond. ... ... ... ... | 16 The Circus, Bath. |
| *Chambers, E. R. ... ... ... ... ... ... ... ... | 9 Clifton Park, Clifton. |
| *Charles, J. R., M.A., M.D., B.C. Cantab. | Deepholm, Clifton Park, Clifton. |
| *Chitty, H., M.S. Lond. ... ... ... ... ... | 46 Pembroke Road, Clifton. |
| *Christofferson, P. E., M.B., Ch.B. Brist. ... | 8 Lansdown Place, Clifton. |
| *Claremont, L. E. ... ... ... ... ... ... ... | 5 Rodney Place, Clifton. |
| *Clarke, R. C., *O.B.E.*, M.B., Ch.B. Brist. ... | 29 Victoria Square, Clifton. |
| *Coates, Vincent M., *M.C.*, M.A., M.D., B.C. Cantab. .. ... ... ... ... ... ... | 10 The Circus, Bath. |
| Connellan, P. S. ... ... ... ... ... ... ... ... | 12 Bloomsbury Street, London, W.1. |
| *Coombs, Carey, M.D. Lond. ... ... ... ... | 3 Pembroke Road, Clifton. |
| *Cooper, William Russell ... ... ... ... | 13 Westfield Park, Redland, Bristol. |
| *Corfield, C., M.D. Durh. ... ... ... ... | 5 Kensington Place, Clifton. |
| Cossham, W. R., M.D., C.M. Aberd.... ... ... | Wellesley House, Cirencester. |
| Cridland, A. B. ... ... ... ... ... ... ... ... | Salisbury House, Chapel Ash, Wolverhampton. |
| *Cross, F. R., M.B. Lond., LL.D. Brist. ... | Worcester House, Clifton. |
| *Crossman, F. W., M.B. Durh. ... ... ... ... | White's Hill, Hambrook, near Bristol. |

| | |
|---|---|
| *Dacre, John ... ... ... ... ... ... ... ... ... | 14 Eaton Crescent, Clifton. |
| *Davies, D. S., M.D. Lond., D.P.H. Cantab. ... | 6 Lansdown Place, Clifton. |
| *Davies, E. D. D. ... ... ... ... ... ... ... ... | Standish House, Stonehouse, Glos. |
| Devine, H., *O.B.E.*, M.D. Lond. ... ... ... | Corporation Mental Hospital, Portsmouth. |
| *Dix, C. ... ... ... ... ... ... ... ... ... ... ... | 11 Victoria Square, Clifton. |
| *Dixon, Helen M., M.B., B.Ch. Brist. ... ... | 10 Whatley Road, Clifton. |
| *Dixon, T. B. ... ... ... ... ... ... ... ... ... | 11 Pembroke Road, Clifton, Bristol. |
| *Drapes, T. L., M.B., B.C., B.A. Cantab. ... | St. Maur, Beaufort Square, Chepstow. |
| *Duerden, J. R., M.B., Ch.B. Brist. ... ... | 149 Bell Hill Road, St. George, Bristol. |
| *Dykes, A. L., M.D. Lond. ... ... ... ... ... | 12 St. Julian Road, Bristol. |
| Dymoke, F. ... ... ... ... ... ... ... ... ... | 21 Cotham Road, Bristol. |

| | |
|---|---|
| *Edgeworth, F. H., M.D., B.C., M.A. Cantab., D.Sc. Lond. ... ... ... ... ... ... ... ... | 13 Richmond Hill, Clifton. |
| *Eglinton, J. H. C. ... ... ... ... ... ... ... | Street, Somerset. |
| *Elkington, G. W., M.B., B.S. Lond. ... ... | Bruton, Somerset. |
| Elliot Bros. Ltd. ... ... ... ... ... ... | *Australian Pharmaceutical Notes & News*, O'Connell Street, Sydney, N.S.W., c/o Grimwade, Ridley & Co., 124 Minories, E.C. |
| Elliott, F. Percy, M.B., C.M. Aberd. ... ... | 113 Grove Rd., Walthamstow, London, E.17. |
| *Elliott, W. H. A., M.B., B.S. Lond. ... ... | 1 Upper Belgrave Road, Clifton. |
| *Every-Clayton, L. E. V., M.D. Lond. | The Quarry, Mullion, Cornwall. |
| | |
| *Faill, C. J. C. ... ... ... ... ... ... ... ... | 19 Portland Square, Bristol. |
| *Fallon, Col. J. ... ... ... ... ... ... ... ... | 35 Cavendish Road, Henleaze, Bristol. |
| Farnfield, W. W. ... ... ... .. .. ... ... | Clive Vale, Gillingham, Dorset. |
| *Fawn, G. F. ... ... ... ... ... ... ... ... ... | 6 Oakfield Road, Clifton. |
| *Fells, A., M.B., C.M. Ed. ... ... ... ... ... | 6 Elton Road, Clifton. |
| *Firth, J. L., M.D., M.S. Lond. ... ... ... ... | 8 Victoria Square, Clifton. |
| *Flemming, A. L., M.B., Ch.B. Brist. ... ... | 48 Pembroke Road, Clifton. |
| *Flemming C. E. S. ... ... ... ... ... ... | Manvers House, Bradford-on-Avon. |
| *Forrester-Brown, Maud, M.D., M.S. ... | 22 Coombe Park, Bath. |
| *Foss, E. V., M.D., Ch.B. Brist. ... ... ... | Summer Hill House, St. George, Bristol. |
| *Fraser, A. D., M.A., M.B., Ch.B. Glasg. ... | 4 Alexandra Road, Clifton. |
| | |
| *Garden, R. R., M.A., M.B., B.Ch. ... ... ... | 9 Harley Place, Clifton. |
| Garland, E. B., M.B., C.M. Ed. ... ... ... | 114*a* Hampton Road, Redland, Bristol. |
| *Gee, C. A. H., M.B., Ch.B. Brist. ... ... ... | Portbury, Nr. Bristol. |
| *Gibbs, A. N. Godby ... ... ... ... ... ... ... | 30 Whiteladies Road, Clifton. |
| *Glenny, E. T., M.B., B.S. Lond. ... ... ... | 102 Pembroke Road, Clifton |
| *Golding, Madge E., M.B., Ch.B. Brist. ... | 76 Cranbrook Road, Redland, Bristol. |
| *Gordon, R. G. M.D., B.Sc. Ed. ... ... ... | 9 The Circus, Bath. |
| *Gornall, W. A., M.B., Ch.B. Brist. ... ... | 23 Windsor Road, St. Andrew's Park, Bristol. |
| *Green, T. A., *D.S.O.*, M.D., C.M. Ed. ... ... | 33*a* Whiteladies Road, Clifton. |
| Griffiths, Cornelius A. ... ... ... ... | 35 Newport Road, Cardiff. |
| *Griffiths, J. S. ... ... ... ... ... ... | Redland Park House, Bristol. |
| *Griffiths, S. J. H., M.B., Ch.B. Brist. ... | Bristol General Hospital. |
| *Groves, E. W. Hey., M.D., M.S. Lond.... ... | 25 Victoria Square, Clifton. |
| | |
| *Hadfield, G., M.D. Lond. ... ... ... ... | Bristol General Hospital. |
| *Hailes Clements, D. G., M.D., C.M. Ed. | 2 Richmond Park Road, Clifton. |
| *Hall, E. George, M.B. Lond. ... ... ... ... | 42 Apsley Road, Clifton, Bristol. |
| *Hall, I. Walker, M.D., Ch.B. Vict. ... ... | Shortwood Lodge, Pucklechurch, Glos. |
| *Ham, C. I., M.B., Ch.B. Brist. ... ... ... ... | Bristol Royal Infirmary. |
| *Harris, H. Elwin, B.A., M.B. Cantab. ... ... | 13 Lansdown Place, Clifton. |
| *Harris, H. E., M.A., M.B., B.Ch. Cantab. ... | 13 Lansdown Place, Clifton. |
| *Harsant, W. H. ... ... ... ... ... ... ... | Tower House, Clifton Down Road, Clifton. |
| *Harty, J. P. I., M.B., B.Ch., B.A.O. R.U.I.... | Deepholm, Clifton. |
| *Hector, F., M.D. Lond. ... ... ... ... ... | Ham Green Hospital, Pill. |
| *Herapath, C. E. K., *M.C.*, M.D. Lond. ... ... | Ormlie, Clifton. |
| Hospital, Bristol General (*Secretary*, T. W. Gregg). | |
| *Houghton, L. M., M.B., B.S. Lond. ... ... | 74 Church Road, Redfield, Bristol. |
| Hughes, D., M.B., B.Ch., B.A.O. N.U.I. ... | 12D Cotham Road, Bristol. |
| *Hunter, F. S. ... ... ... ... ... ... ... | c/o Messrs. J. Wright & Sons Ltd., Publishers, Bristol. |

| | |
|---|---|
| *Iles, A. E., *O.B.E.*, M.B., B.S. Lond. ... ... | 17 Victoria Square, Clifton. |
| Infirmary, Bristol Royal (*Secretary*, E. C. Smith). | |
| Ingle, C. Durban ... ... ... ... ... ... ... | Somerton, Somerset. |
| *Jackman, W. A., M.B., Ch.B. Brist.... ... ... | 15 Mortimer Road, Clifton. |
| *Johnson, R. G., M.B. Lond. ... ... ... ... | 119 Redland Road, Bristol. |
| *Joll, C. A., M.B., M.S. Lond. ... ... ... ... | 64 Harley Street, London. |
| *Kemm, N. F., M.B., B.S. Lond.... ... .. ... | 12 Duchess Road, Clifton. |
| *Kennedy, W. P., M.D. Dub. ... ... ... ... | 3 Gay Street, Bath. |
| *Kerfoot, Stanley J. ... ... ... ... ... ... | 194 Redland Road, Bristol. |
| *Kingston, S. H., M.B., Ch.B. Brist. .. ... | 3 Whiteladies Road, Clifton. |
| *Kyle, H. G., M.A., M.D. B.Ch. Oxon. ... ... | 31 Westbury Road, Bristol. |
| *Lace, F. ... ... ... ... ... ... ... ... ... | 5 Gay Street, Bath. |
| *Lavington, C C., M.B., B.S. Durh. ... ... | 1 Burlington Road, Redland, Bristol. |
| *Lees, E. Leonard, M.D., C.M. Ed. ... ... ... | 22 Richmond Hill, Clifton. |
| *Legat, R. Eddowes, M.B., C.M. Ed. ... ... | Murray House, Staple Hill, Bristol. |
| Leigh, W. W. ... ... ... ... ... ... ... ... | Llansamnor House, Cowbridge, Glam. |
| *Leonard, R. C., M.D. Durh. ... ... ... ... | Bower Ashton, Bristol. |
| *Lester, Muriel A., M.B., B.S. ... ... ... | 44 Clifton Park Road, Bristol. |
| *Levis, J. S., *M.C.*, M.B., B.Ch., N.U.I. ... | 20 Gay Street, Bath. |
| *Linton, Marion S., M.B. Lond. ... ... ... | 21 Oakfield Road, Clifton. |
| *Lister, A. E. J., M.B., B.S. Lond. ... ... | 86 Pembroke Road, Clifton. |
| *Lister, L. Margaret ... ... ... ... ... | 12 All Saints Road, Clifton. |
| *Logan, H. B. ... ... ... ... ... ... ... ... | Elmwood, Aberdeen Road, Clifton. |
| *Lucas, J. J. S., M.D. Lond. ... ... ... ... ... | Highgrove, Wells Road, Totterdown, Bristol. |
| *Lysaght, A. C. ... ... ... ... ... ... ... | 8 Clifton Hill, Bristol. |
| *Macdonald, P. T. T., M.A., M.B., Ch.B. ... | 43 Regent Street, Kingswood, Bristol. |
| *McKenzie, W. G., *M.C.* ... ... ... ... ... | 101 Coronation Road, Bristol. |
| Mackinnon, Charles, M.B., C.M., M.A. Glasg. | Davaar, Cirencester. |
| *McLannahan, J. G. ... ... ... ... ... ... | The Mount, Stonehouse. |
| Maclean, Sir Ewen J., M.D., C.M. Ed. ... | 12 Park Place, Cardiff. |
| *MacLeod, Donald, M.B., C.M. Ed. ... ... | 84 Redland Road, Redland, Bristol. |
| *MacLeod, G., *M.C.*, M.D., Ch.B. ... ... ... | "Wargrave," Clevedon. |
| *MacWatters, J. C., *M.B.E.* ... ... ... ... | Almondsbury, near Bristol. |
| Marle, S.... ... ... ... ... ... ... ... ... | Newbridge Road, Bath. |
| Marshall, Arthur L., M.B., B.A. Cantab. ... | Raveningham, Norwich. |
| Mather, J. S., M.B., C.M. Aberd. ... ... ... | Lawrence Hill House, Bristol. |
| *Mathew, W. E., M.D., C.M. Toronto. ... ... | 85 Coldharbour Road, Redland. |
| *Mayes, F. J. A. ... ... ... ... ... ... ... | 79 Pembroke Road, Clifton. |
| Miall, C. L'O. ... ... ... ... ... ... ... | Elm Hayes, Paulton, near Bristol. |
| Middleton-West, S.H., *M.C.*, M.B., Ch.B. Vict. ... ... ... ... | 28 Percival Road, Clifton. |
| *Milling, T., M.B., Ch.B. Belfast. ... ... ... | 1 Vicarage Road, Southville. |
| *Moore, C. A., M.B., M.S. Lond. ... ... ... | 56 St. Paul's Road, Clifton. |
| *Moore, L. A. ... ... ... ... ... ... ... | Hillsborough, Cotham Park, Bristcl. |
| *Morris, A. G. ... ... ... ... ... ... ... | 18 Westbury Park, Westbury-on-Trym. |
| Morris, L. N., M.B., Ch.B. Brist. ... ... ... | 24 All Hallows Road, Upper Easton, Bristol. |
| *Morton, C. A., *O.B.E.* ... ... ... ... ... ... | 14 Vyvyan Terrace, Clifton. |
| *Mumford, W. G., M.B. Lond. ... ... ... ... | 18 The Circus, Bath. |
| Mundy, G. S., M.B., B.Ch. Brist. ... ... ... | Southmead Hospital, Bristol. |
| *Murray, W. ... ... ... ... ... ... ... ... | Fairfield, Walton, Clevedon. |
| *Myles, G. T. ... ... ... ... ... ... ... ... | 7 Apsley Road, Clifton. |

*Neild, Newman, M.B., B.Ch. Vict. ... ... ... 9 Richmond Hill, Clifton.

*Newnham, W. H. C., M.B., M.A. Cantab. ... 3 Lansdown Place, Victoria Square, Clifton.

*Nichols, F. C., *M.C.*, M.B., Ch.B. Brist. ... 38 Whiteladies Road, Clifton.

*Nixon, J. A., *C.M.G.*, M.D. Cantab. ... ... 7 Lansdown Place, Clifton.

*Nott, Winifred, M.B., Ch.B. Brist. ... ... "Fassiefern," Upper Cranbrook Road, Redland, Bristol.

*Ormerod, H. Lawrence, M.D., B.Ch., R.U.I. 2 Henleaze Road, Westbury-on-Trym, Bristol.

Orr-Ewing, H. J. *M.C.* M.D. Lond. ... ... English Mission Hospital, Jerusalem.

*Packer, M. E. J., M.B., Ch.B. Brist. ... ... 4 Effingham Road, St. Andrew's Park, Bristol.

*Parker, George, M.D., M.A. Cantab. ... ... 14 Pembroke Road, Clifton.

Parkes, J. A., M.B., Ch.B. Liverpool ... ... 8 South Road, Redland, Bristol.

Parkinson, Major G. S., *D.S.O.*, R.A.M.C.... R.A.M.C. Mess, Grosvenor Place, London, S.W. 1.

Parsons, Sir J. H., M.B., B.S., D.Sc. Lond.... 54 Queen Anne Street, London, W.

*Phillips, J. R. P., *C.B.E.* ... ... ... ... ... Northwoods, Winterbourne, nr. Bristol.

*Phillips, P., M.B., Ch.B. Brist. ... ... ... Southmead Hospital, Bristol.

*Pollard, J., M.B., B.Ch., B.A.O. ... ... ... 52 Cumberland Road, Bristol.

*Popper, O. O., M.B., Ch.B. Ed. ... ... ... 14 Victoria Square, Clifton.

Powell, J. J., M.D. Lond. ... ... ... ... ... 2 Gloucester Road, Bishopston, Bristol.

*Pridie, H. H., M.B., C.M. Ed. ... ... ... 17 Oakfield Road, Clifton.

*Rayner, D. C.... ... ... ... ... ... ... ... ... 9 Lansdown Place, Clifton, Bristol.

*Renton, R. A. S., *M.C.*, M.D. Durh.... ... ... Emsworth House, Clevedon.

*Robb, J. J. ... ... ... ... ... ... ... ... ... 25 Victoria Square, Clifton.

*Robertson, D., M.B., Ch.B. Ed. ... ... ... 205 Wells Road, Knowle, Bristol.

*Rogers, Beatrice ... ... ... ... ... ... ... 16 Richmond Hill, Clifton.

*Rogers, B. M. H., M.D., B.Ch., B.A. Oxon. ... 14 Mortimer Road, Clifton.

*Rolfe, R., B.A., M.B., B.C. Cantab. ... ... Park House, St. Andrews Road, Avonmouth.

*Rowley, A. T.... ... ... ... ... ... ... ... ... Wrington.

*Rutherford, J. M., M.B., C.M. Ed. ... ... Brislington House, near Bristol.

Salisbury, Walter, M.D., M.S. Lond. ... 19 The Drive, Northampton.

Savage, W. G., M.D., B.Sc. Lond. ... ... ... "Bourn," 7 Trewartha Park, Weston-super-Mare.

*Scott, H. H. ... ... ... ... ... ... ... ... ... 78 Old Market Street, Bristol.

*Shaw J. E., M.B., C.M. Ed. ... ... ... ... 23 Caledonia Place, Clifton.

*Short, A. R., M.D. ... ... ... ... ... ... 69 Pembroke Road, Clifton.

Shepherd, H. L., M.B., Ch.M. Brist. ... 29 Victoria Square, Clifton.

*Smith, G. Cockburn, M.D. Brux. ... ... ... 12 South Road, Newton Abbot.

*Short, L. J., M.D., B.S. Lond., M.D. Brist.... Maywood, Atlantic Road South, Weston-super-Mare.

*Smith, W. Alexandef, M.B., M.A. Oxon. ... 70 Pembroke Road, Clifton.

*Smyth, Richard, M.A., M.D. Dub. ... ... Burnley, Westbury-on-Trym, Bristol.

*Smythe, H. J. D., *M.C.*, M.B., M.S. ... ... 15 Eaton Crescent, Clifton.

*Spoor, A., M.B., B.Ch. Cantab.... ... ... ... The Hydro, Bristol.

*Staniland, M. F. ... ... ... ... ... ... ... 255 Stapleton Road, Bristol.

| | |
|---|---|
| *Statham, R. S. S., *O.B.E.*, M.D., M.Ch. Brist. | 24 College Road, Clifton. |
| *Stewart, A. L., D.M., D.Ch. Brux. ... ... | 31 Howard Road, Southville, Bristol. |
| *Stock, W. S. V., M.B. Lond. ... ... ... | 1 Mortimer Road, Clifton. |
| *Strover, H. W. M., *O.B.E.*, M.B., Ch.B. Aberd. ... ... ... ... ... ... ... ... | 24 Gloucester Road, Bishopston, Bristol. |
| *Symes, J. O., M.D. Lond. ... ... ... ... | 71 Pembroke Road, Clifton. |
| | |
| *Tasker, D. G. C., M.B., M.S. Lond. ... ... | 16 All Saints Road, Clifton. |
| Taylor, A. L. ... ... ... ... ... ... ... | The Royal Infirmary, Bristol. |
| *Taylor, H. N., M.A. Cantab., M.D. Dub.... | Hillside, Ebbw Vale. |
| *Temple, G. H., M.B., C.M. Ed. ... ... ... | Ailanthus, Weston-super-Mare. |
| *Terry, C. H., M.A., M.B., B.Ch. Oxon. ... | 15 The Circus, Bath. |
| Thin, James ... ... ... ... ... ... ... ... | 54 & 55 South Bridge, Edinburgh. |
| *Thomas, J. D., M.B. Cantab. ... ... ... | Northwoods, Winterbourne. |
| *Todd, A. T., *O.B.E.*, M.B., Ch.B. Ed. ... | Clayton, Clifton Park, Clifton. |
| *Trist, J. R. R., *M.C.* ... ... ... ... ... ... | 120 Henleaze Road, Clifton. |
| *Tryon, V., M.B., Ch.B. Brist. ... ... ... | 3 Harley Place, Clifton. |
| | |
| *Vickery, W. H. ... ... ... ... ... ... ... | 29 Trewartha Park, Weston-super-Mare. |
| | |
| *Walker, Cyril H., M.B., B.A. Cantab. | 8 Oakfield Road, Clifton. |
| *Wallace, John, *O.B.E.*, M.B., C.M. Ed. | 10 Knightstone Road, Weston-super-Mare. |
| *Walters, C. F. ... ... ... ... ... ... ... | 5 Mortimer Road, Clifton. |
| *Wanklyn-James, C. W., *O.B.E.* ... ... | 3 Mortimer Road, Clifton. |
| *Warren, R., M.A., M.D. Oxon. ... ... ... | Ellenborough Hall, Weston-super-Mare. |
| *Waterhouse, R., M.D.Lond. ... ... ... | 25 The Circus, Bath. |
| *Watson-Williams, E., *M.C.*, B.A. Cantab. | 12 Victoria Square, Clifton. |
| *Weston, B. A. A., M.B., B.Ch. Brist. ... | 30 Cotham Grove, Bristol. |
| *Weatherhead, E., M.B., B.Ch. Cantab. ... | 12 Sion Hill, Clifton. |
| *Whicher, A. H. ... ... ... ... ... ... ... | 115 Queen's Road, Clifton. |
| *White, E. B. C. ... ... ... ... ... ... ... | Mental Hospital, Fishponds, Bristol. |
| Whitehouse, H. Beckwith, M.S. Lond. ... | 52 Newhall Street, Birmingham. |
| Wigan, C. A., M.D. Durh., J.P. ... ... ... | Deepdene, Portishead. |
| Wigmore, J., M.D., R. U. I. ... ... ... | 16 Queen Square, Bath. |
| *Willcox, J. W. J., M.B., B.S. Lond. ... | Glastonbury. |
| Willcox, R. Lewis ... ... ... ... ... ... | 97 London Road, Salisbury. |
| Williams, S. R., M.B. Lond. ... ... ... | Buckfastleigh, Devon. |
| *Wills, W. K., *O.B.E.*, M.B., B.C. Cantab. | 19 Whiteladies Road, Clifton. |
| *Wood, Duncan ... ... ... ... ... ... ... | 5 Eaton Crescent, Clifton. |
| *Wood, W. V., *M.C.*, B.A. Cantab. ... ... | Henley Lodge, Yatton, Som. |
| *Wright, A. J., M.B., B.S. Lond. ... ... | 2 Clifton Park, Clifton. |
| | |
| *Zealand, L., M.D. Man. ... ... ... ... | Brecon Lodge, Westbury-on-Trym, Bristol. |

